# Divergent functions of NLRP3 inflammasomes in cancer: a review

**DOI:** 10.1186/s12964-023-01235-9

**Published:** 2023-09-15

**Authors:** Alireza Shadab, Mohamad Mahjoor, Mohammad Abbasi-Kolli, Hamed Afkhami, Parisa Moeinian, Amir-Reza Safdarian

**Affiliations:** 1 Department of Immunology, School of Medicine, Semnan University of Medical Sciences, Semnan, Iran; 2 Iran University of Medical Sciences, Deputy of Health, Tehran, Iran; 3 Cellular and Molecular Research Center, Qom University of Medical Sciences, Qom, Iran; 4 Department of Immunology, Faculty of Medicine, Iran University of Medical Sciences, Tehran, Iran; 5 Department of Medical Genetics, Faculty of Medical Sciences, Tarbiat Modares University, Tehran, Iran; 6 Nervous System Stem Cells Research Center, Semnan University of Medical Sciences, Semnan, Iran; 7 Department of Medical Microbiology, Faculty of Medicine, Shahed University, Tehran, Iran; 8 Department of Medical Genetics and Molecular Biology, School of Medicine, Iran University of Medical Sciences, Tehran, Iran; 9 Immunology Board for Transplantation and Cell-Based Therapeutics (Immuno TACT), Universal Scientific Education and Research Network (USERN) Chicago, Chicago, IL USA; 10 Department of Immunology and Microbiology, Faculty of Veterinary Medicine, Tehran University, Tehran, Iran

**Keywords:** Inflammasome, Caspase, Pyroptosis, Tumor, Immunotherapy, Immunosuppression, Metastasis, NLRP3

## Abstract

The cancer is a serious health problem, which is The cancer death rate (cancer mortality) is 158.3 per 100,000 men and women per year (based on 2013–2017 deaths). Both clinical and translational studies have demonstrated that chronic inflammation is associated with Cancer progression. However, the precise mechanisms of inflammasome, and the pathways that mediate this phenomenon are not fully characterized. One of the most recently identified signaling pathways, whose activation seems to affect many metabolic disorders, is the “inflammasome” a multiprotein complex composed of NLRP3 (nucleotide-binding domain and leucine-rich repeat protein 3), ASC (apoptosis associated speck-like protein containing a CARD), and procaspase-1. NLRP3 inflammasome activation leads to the processing and secretion of the proinflammatory cytokines interleukin-1β (IL-1β) and IL-18. The goal of this paper is to review new insights on the effects of the NLRP3 inflammasome activation in the complex mechanisms of crosstalk between different organs, for a better understanding of the role of chronic inflammation in cancer pathogenesis. We will provide here a perspective on the current research on NLRP3 inflammasome, which may represent an innovative therapeutic target to reverse the malignancy condition consequences of the inflammation.

Video Abstract

**Supplementary Information:**

The online version contains supplementary material available at 10.1186/s12964-023-01235-9.

## Inflammasomes & cancer: an overview

Cancer progression is a complicated procedure that involves both tumor cell-intrinsic and -extrinsic signals that promote cellular transition, unregulated growing, invasion, and metastasis, and inflammation that leads to tumor escape from immunological clearance that have a significant role in malignancies [1, 2]. primary protective layer is comprised of inflammatory innate immune responses since they easily detect "danger" signals through pattern recognition receptors (PRRs) like Toll-like receptors (TLR), C-type lectin receptors (CLR), RIG-I-like receptors (RLR) and one of the important types of PRRs is Nod-like receptors (NLRs). There are 14 members of this protein subfamily in humans (called NLRP1 to NLRP14). NLRP3 and NLRP4 are examples of inflammasome receptors that offer adjuvant effects to activate the adaptive immunity pathway. However, there are other NLRPs that can also form inflammasomes, such as NLRP1, NLRP2, NLRP6, NLRP7, and NLRP12 [3].

Inflammasomes are made up of the NOD-like receptor (NLR) family, the adapter apoptosis-associated speck-like protein containing a caspase recruitment domain [4], and the effector protease caspase-1. The NLR family is classified as either NLRP or NLRC depending on whether the N-terminus contains a pyrin domain (PYD) or a caspase activation and recruitment domain [4, 5]. Assembling an inflammasome is a well-known function of the NLRs including NLRP1 (mouse NLRP1b), NLRP3, and NLR family apoptosis inhibitory protein (NAIP)-NLRC4 [6]. Other NLR sensors, such as NLRP6, NLRP7, NLRP9, NLRP12, NLRC3, and NLRC5, as well as non-NLR sensors like interferon gamma-inducible protein 16 and retinoic acid-inducible gene I, may structure inflammasome complexes in context-dependent forms [7–9]. The assembly of these biomolecules’ triggers caspase-1, which is responsible in the maturity of the proinflammatory cytokines interleukin-1 (IL-1β) and IL-18 into bio—active forms, as well as the cleavage of gasdermin D (GSDMD), which promotes pyroptotic apoptosis (pyroptosis) [6, 10–12]. Although IL-1β and IL-18 are the major cytokines promoted by the NLRP3 inflammasome activation, they are not exclusive to this pathway. IL-1β and IL-18 can also be produced by other inflammasomes, such as AIM2 or NLRC4 [13], or by non-inflammasome mechanisms, namely TLRs or RIPK3 [14]. Conversely, the NLRP3 inflammasome can also regulate other molecules besides IL-1β and IL-18, including gasdermin D (GSDMD), which mediates pyroptosis [15], or mitochondrial DNA (mtDNA), which induces type I interferon response [16]. Therefore, it is important to distinguish between the effects of IL-1β/IL-18 and the effects of NLRP3 inflammasome in cancer biology. For example, some studies have shown that blocking IL-1β or IL-18 can inhibit tumor growth or metastasis [17, 18], while others have proven that inhibiting NLRP3 inflammasome can enhance tumor growth or metastasis [19, 20]. These discrepancies may reflect the different roles of IL-1β/IL-18 and NLRP3 inflammasome in different cancer types or stages.

The NLRP3 inflammasome is a multiprotein complex that senses various endogenous and exogenous stimuli and activates caspase-1, which in turn cleaves pro-IL-1β and pro-IL-18 into their mature forms. These cytokines play prominent role in inflammation, immunity, and tumorigenesis [16]. However, the NLRP3 inflammasome is not only a source of IL-1β and IL-18, but also a regulator of other signaling pathways, such as NF-κB, pyroptosis, autophagy, and oxidative stress [16]. Therefore, the NLRP3 inflammasome has complex and context-dependent effects on cancer development and progression. The NLRP3 inflammasome is more important than the other inflammasomes in cancer because it has a dual role in cancer progression and regression [21]. In fact, the role of NLRP3 inflammasome in cancer is complex and context-dependent. For instance, In gastric cancer, the NLRP3 inflammasome enhances cell differentiation and induces IL-1β production, which activates NF-κB and JNK signalling, leading to proliferation, invasion, and cancer development or In breast cancer, the NLRP3 inflammasome and IL-1β production promote the infiltration of myeloid cells, providing an inflammatory microenvironment that promotes breast cancer progression [22]. On the other hand, the NLRP3 inflammasome can inhibit tumorigenesis in colitis-associated cancer [23]. Moreover, The NLRP3 inflammasome mediates pyroptosis, which inhibits tumor development in colorectal cancer (CRC) or colitis-associated cancer which is a major complication of inflammatory bowel diseases [21]. Therefore, more research is needed to understand the mechanisms and implications of NLRP3 inflammasome activation in different cancer settings, and we focus on the role of NLRP3 inflammasome in cancer in the present review.

**Fig. 1:**
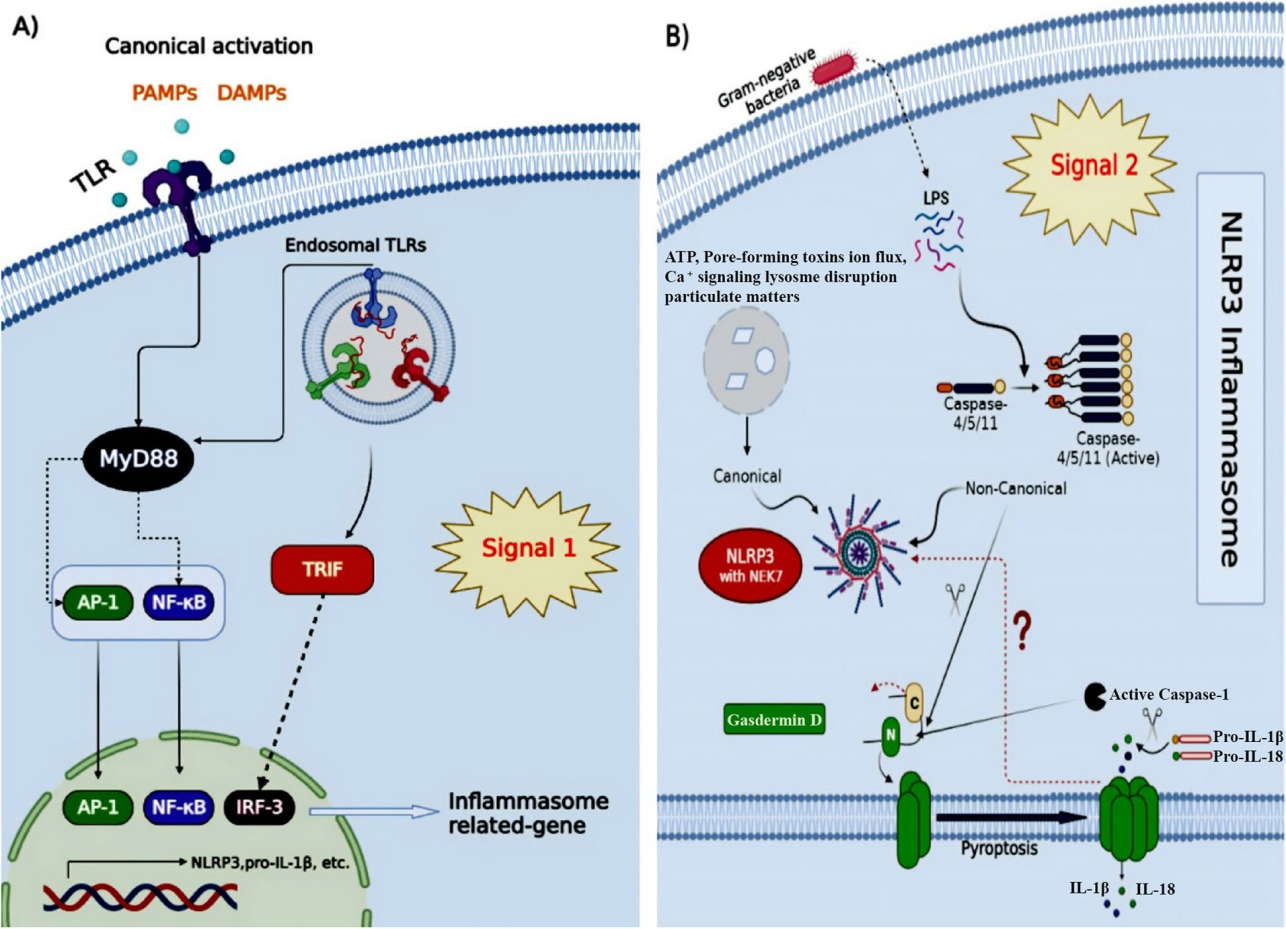
Overview of NLRP3 inflammasome priming and activation. NLRP3 inflammasome activation involves two steps, i.e., Signal 1 (priming) and Signal 2 (protein complex assembly). The NLRP3 inflammasome requires two steps for activation, referred to as Signal 1 (priming) and Signal 2 (protein complex assembly). The canonical pathway for activation involves the priming signal inducing the transcription of inflammasome components, and the activating signal subsequently causing the formation of the complex. Different families of receptors (NLR, ALR, and PYRIN) recognize various MAMPs or DAMPs to initiate this process. Caspase-1 is essential in this process as it breaks down pro-inflammatory cytokines into their active forms and GSDMD into a N-terminal pore-forming domain that induces pyroptosis. The non-canonical route is initiated by LPS or phospholipids binding directly to caspase-4/-5 in humans

NLRP3 is a cytoplasmic PRR with a tripartite domain organization consisting of a carboxy-terminal leucine-rich repeat (LRR) domain with autoinhibitory activities and signal recognition capabilities, a central nucleotide-binding domain (NACHT) as well as NOD with ATPase activity and mediates self-oligomerization, and an amino-terminal pyrin domain (PYD) that engages a [4, 24]. The greatest studied inflammasome complex is the NLRP3 (NOD-, LRR-, and pyrin-domain containing protein 3), which is triggered by a range of stimuli [25]. It is commonly recognized that NLRP3 inflammasome activation is controlled in two steps: transcriptional and posttranslational priming (i) and assembly via different pathways in response to various kinds of external pathogen-derived or endogenous threats molecules [26]. The priming stage causes nuclear factor-B (NF-kB)-dependent increase of NLRP3 and pro-IL-1 expression as well as further post—translational modification to reduce NLRP3 activation level (PTMs). The second stage is the identification of NLRP3 activating agent, which causes NLRP3 stimulation and the development of an inflammasome. Often these pattern recognition receptors (PRR) are unique for one or a few relevant pathogen-associated molecular patterns (PAMPs) or damage-associated molecular patterns (DAMPs). Toxins, crystals, aggregates (Beta-amyloid), extracellular ATP, and hyaluronan are just a few instances of the impressive list of bacterial, viral, and fungal PAMPs and endogenous DAMPs which may activate the NLRP3 [27–29]. In addition, NLRP3 inflammasome components that interact with other proteins and undergo post-translational modification (PTM) allow cells to fully activate the inflammasome assembl [30–33] (Fig. 1).

**Fig. 2:**
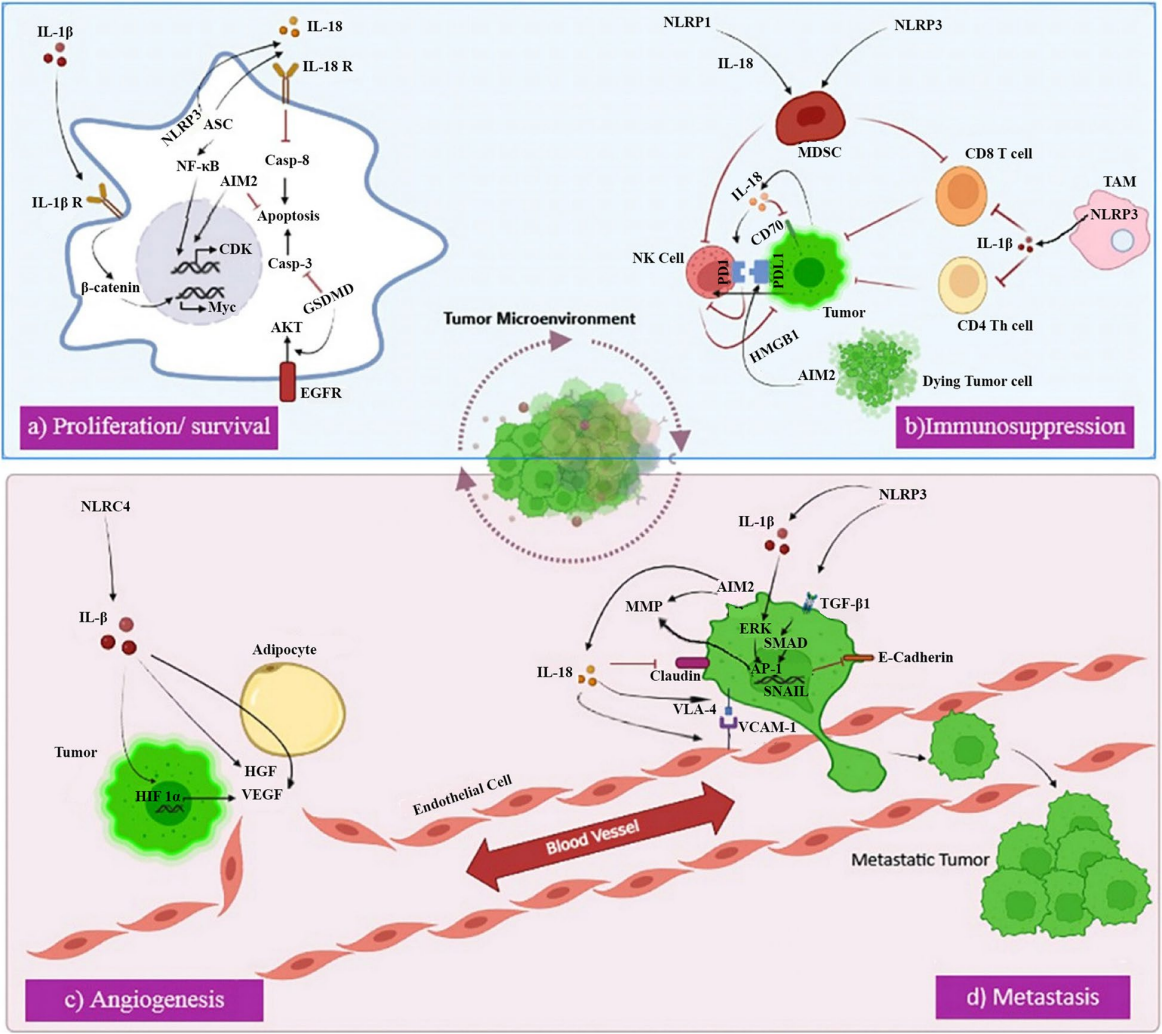
**a** Proliferation: NLRP3-mediated release of IL-18 or IL-1β activates β-catenin which causes the oncogene c-Myc to be expressed, resulting in an increase in proliferation of lymphoma cells. In gastric epithelial cancer cells, ASC induces IL-18 production and NF-ΚB activation, leading to cellular proliferation. Gasdermin-D (GSDMD) promotes AKT signaling in non-small cell lung cancer. **b** Immunosuppression: NLRP1-mediated IL-18 production in multiple myeloma stimulates the generation of myeloid-derived suppressor cells (MDSCs) in the immune niche, inhibiting CD8 + T-cells and NK cells and resulting in tumorigenesis. NLRP3-mediated IL-1β from TAMs suppresses anti-tumor immunity of CD4 + Th1 cells and CD8 + T-cells. HMGB1 released from disrupted mitochondrial iron metabolism through AIM2 increases PDL1 expression in pancreatic ductal adenocarcinoma. IL-18 enables immune escape of gastric cancer cells by upregulating PD1 on NK cells and downregulating CD70 on tumor cells, which reduces cytotoxicity of NK cells and induces tumor specific T cell memory. **c** Angiogenesis: NLRC4 inflammasome mediated release of IL-1β acts on adipocytes to induce vascular endothelial growth factor (VEGF) production. In tumor cells, IL-1β stimulates secretion of hepatocyte growth factor (HGF) and hypoxia-inducible factor-1 (HIF1α) expression to transcriptionally regulate VEGF production. **d** Metastasis: NLRP3 increases EMT by enhancing TGF β 1 mediated Smad signaling which decreases E cadherin expression in squamous cell carcinoma and gastric carcinoma. AIM2 increases MMPs which allows for invasion of cutaneous squamous cell carcinoma. IL 1β increases activator protein (AP 1) transcriptional activity that increases MMPs for invasiveness of breast ductal cancer cells. Tumor derived IL 18 induces vascular cell adhesion molecule-1 (VCAM 1) expression in HSECs and very late antigen 4 (VLA 4) in melanoma cells that facilitates VCAM 1 dependent melanoma cell adhesion to HSECs

Canonical NLRP3, a non-ligand binding sensor, is reported to react to cellular irregularities identified by lysosomal rupture, potassium efflux, mitochondrial DNA or disruption, calcium influx, or reduction in cellular cAMP levels, all induced by numerous PAMPs and DAMPs – for example toxins, pathogens, crystalline compounds, and metabolites [34]. It has been demonstrated that the serine/threonine protein kinases NEK7 and MAP kinase TGF-beta activated kinase-1 (TAK1) regulate the activity of NLRP3 [35–37]. Furthermore, Z-DNA-binding protein controls the activation of the NLRP3 inflammasome during influenza A virus infection [38]. It is unclear that direct structural recognition or ligand binding are involved in the broad range of Recruitment and activation. NLRP3 may play a role as a sensor for homeostasis-altering molecular processes (HAMPs) [39] (Fig. 2).

Human caspases 4 and 5 directly bind to cytoplasmic lipopolysaccharide, which causes non-canonical NLRP3 inflammasome activation as a ligand-binding sensor as well, although the molecular mechanism is unknown [40]. The activity of NLRP3 inflammasome must be rigorously managed, and the entire mechanism of its activation must be explored further.

## Inflammasome pathogenesis

The activity of the inflammasome, a multi-protein complex that amplifies immune reactions to both foreign and endogenous activators, is one of the primary inflammatory processes that result in the occurrence of inflammatory disorders including cancer [10, 41, 42]. Increased NLRP3 inflammasome activity also accelerates up the development of several inflammatory conditions, such as cryopyrin-associated periodic syndrome, arthritis, atherosclerosis, type 2 diabetes, Alzheimer’s disease, and cancers [43–45]. Throughout the last years, the complicated involvement of inflammasomes in tumorigenesis and anticancer responses in malignancies have been uncovered. The fundamental biological characteristics that tumor cells acquire during the cascade of events in tumorigenesis assess tumor hallmarks [46, 47]. Furthermore, it has been well widely recognized that chronic inflammation influences tumorigenesis. In fact, chronic inflammation plays a role in the majority of carcinogenesis phases [48]. Numerous cell types, including fibroblasts, endothelial cells, macrophages, and tumor cells, produce proinflammatory cytokines during this process, which has the ability to encourage the onset, development, and metastasis of various cancers [49]. We provide a summary of the interaction between carcinogenesis and the NLRP3 inflammasome in this article, and discuss their potential as a therapeutic target. Immune cells like macrophages and dendritic cells have been the focus of much of the research on inflammasomes, yet, non-hematopoietic cells can also express and assemble inflammasomes [7].

## Various cancers

The NLRP3 inflammasome is a cytosolic protein complex that regulates inflammation and immunity in response to cellular stress. It activates caspase-1, which leads to the production and release of pro-inflammatory cytokines such as IL-1β and IL-18, as well as inflammatory cell death (pyroptosis). The NLRP3 inflammasome has been implicated in various types of cancer, such as breast, lung, prostate, colorectal, bladder and melanoma. However, its role in cancer is complex and context-dependent, as it can exert both pro-tumorigenic and anti-tumorigenic effects. Therefore, understanding the mechanisms and consequences of NLRP3 inflammasome activation in cancer is crucial for developing novel therapeutic strategies [50].

### Breast cancer

The most frequent cancer in the world is still breast cancer [51–53], and is the most prevalent neoplasm in women and the second leading cause of cancer mortality [54, 55]. Radiotherapy until now a key component of treating This deadly cancer, but de-escalation techniques are now considered the norm [56]. The tumor microenvironment (TME) changes continuously during tumorigenesis as a result of malignant cell mutations, the diverse nature of the microenvironmental composition, and the various stromal cell proportions and states of activation [57]. By infiltrating the TME, releasing cytokines, growth factors, chemokines, and proangiogenic factors, and causing genome instability and immune evasion, inflammation can increase the risk of cancer-promoting cells [58]. Inflammation and immunosuppression work together to promote tumor development [59]. Chronic inflammation, which is caused by abnormal NF-κB or inflammasome activation, is linked to cancer via PRR-mediated cytokine production [60]. Recent research shows that PRRs and their regulators have both positive and negative effects on cancer cells [54]. On the one hand, some PRRs stimulate an anti-tumor immune response, which slows tumor progression [61]. It has recently been demonstrated that increased expression of the NLRP3 inflammasome in human breast Cancer-Associated Fibroblasts (CAFs) is a precursor to cancer progression and metastasis [62]. The NLRP3 inflammasome has been shown to promote breast cancer growth and metastasis by inducing IL-1β secretion, which stimulates angiogenesis, immune evasion, epithelial-mesenchymal transition (EMT), and stemness [63]. However, some studies have also reported that the NLRP3 inflammasome can suppress breast cancer by inducing pyroptosis, a form of inflammatory cell death, or by activating anti-tumor immune responses [64]. Thus, the role of NLRP3 inflammasome in breast cancer may depend on the tumor microenvironment and the subtype of breast cancer. Because of its overexpression in tumor areas and its association with larger tumor size, higher histological grade, and positive node and receptor status, the NLRP3 inflammasome appears to be involved in tumor aggressiveness [62]. This component is involved in a variety of physiological and pathological conditions, and their role in various cancers has recently been highlighted and it play a dual role in cancer. On the one hand, activation of inflammasomes promotes tumor progression by increasing cancer stem cells, myeloid-derived suppressor cells (MDSCs), metastasis, epithelial mesenchymal transition (EMT), and angiogenesis while inhibiting apoptosis [65] and also it can limit tumor cell survival by promoting pyroptosis and supporting tumor suppressors and immune responses [66]. The most well-studied inflammasome involved in cancer development is the NLRP3 [67]. In the TCGA breast cancer dataset, NLRP3 had a positive correlation with survival in all molecular subtypes [68]. Activation of the NLRP3 inflammasome causes abnormal secretion of soluble cytokines, resulting in a favorable inflammatory environment that promotes tumor growth [62]. The NLRP3 inflammasome-dependent release of IL-1β induces the expression and release of immune cells, CD4 + T cells, and IL-22, which has been linked to the initiation and progression of many types of cancer, including breast cancer [69]. In the context of this disease, it has been revealed that IL-1 and NLRP3 were overexpressed in the breast tumor microenvironment concomitant with the increase of MDSCs and TAMs [70]. NLRP3 and IL-1 expression in TAMs, on the other hand, was associated with survival, lymph node invasion, and metastasis in patients with HER2 + breast cancer [71]. Besides, NLRP3 activation and IL-1β secretion have been linked to tumor growth, invasiveness, relapse, and progression in recent studies [72]. In an effort to validate the role of NLRP3 in breast tumor growth, it was discovered that blocking the IL-1β receptor promotes apoptosis and prevents cell cycle progression in cancer cells [73]. nevertheless, in some studies, it has been observed that the inflammasome can also play an antitumor role Inflammasomes play an anti-tumoral role in cancer therapies in response to immunogenic chemotherapy [74]. Inflammasome activation and pyroptosis induction upstream of IL-1β release may make tumors more sensitive to therapy. The most effective examples of therapies used in This aggressive disease targeting the NLRP3 inflammasome are IL-1 signaling pathway inhibitors that block the IL-1α or IL-1β receptor [75]. Direct inflammasome activation within the tumor may be an important mechanism for engaging antitumor immunity because NLRP3 activation causes pyroptotic, immunogenic cell death and the release of pro-inflammatory factors [76]. As abnormal NLRP3 inflammasome activation has been linked to cancer initiation, there is a great deal of clinical interest in the development of potential NLRP3 inflammasome inhibitors [54]. The inclusion of NLRP3-driven inflammation adds additional impetus to such treatment because it has been manifested that NLRP3 activation in various tissues can activate NK cell responses, which may contribute to tumor clearance [77]. Interestingly, in murine invasive breast cancer models, the absence of a functional NLRP3 inhibited tumor growth, and NK cell depletion abolished the anti-tumoral effect independently of IL-1 β and IL-18 effector mechanisms [65]. TAK1 inhibitors' dual function of causing cancer cell death while simultaneously activating NLRP3 makes them a potentially powerful anticancer agent for this Aggressive fatal disease [78]. Also Huang et al. It has just been proven that the tumor suppressor lipid and protein phosphatase PTEN directly interacts with NLRP3 and dephosphorylates it, allowing NLRP3-ASC interaction, inflammasome assembly and activation, and myeloid PTEN to determine chemotherapy responsiveness by promoting NLRP3-dependent antitumor immunity [72].

### Lung cancer erythematosus

For both men and women, lung cancer is the leading cause of cancer death [79]. NSCLC is the most common type of lung cancer and the leading cause of cancer-related death worldwide [80]. Cancer cells interact with their microenvironment, particularly immune cells, resulting in stimulatory or inhibitory effects that result in tumor escape or elimination [80]. TAMs are the most common immune cells found in the tumor microenvironment, promoting tumor growth, invasion, and metastasis.Because tumor cells produce various cytokines and chemokines to adapt the TME to their needs, high serum levels of pro-inflammatory cytokines such as IL-1 β, IL-6, IL-8, IL-12, and IL-18 have been found in several types of cancer, including lung cancer [81]. If the cytokine production continues, it can lead to chronic inflammation, has been linked to carcinogenesis and tumorigenesis, including cancer cell initiation and promotion, tumor progression, angiogenesis, and invasion [82]. Other research has indicated that cancer-related inflammation may promote tumor growth and metastasis [83]. Inflammasomes are important components of innate immune responses and can have tumor-suppressive or oncogenic functions, but their role in lung cancer is unclear and The field of inflammasome mediators and inflammasome activation in human lung cancer is still relatively unknown [84] and it can play an important role in the development and pathogenesis of lung cancer because the lungs are a tissue niche that is prone to inflammation due to its exposure to external substances [81]. Furthermore, numerous studies show that the NLRP3 promotes melanoma cell lung metastasis and supports HCC metastasis in mouse lung metastasis models. On the other hand, inhibiting NLRP3 inflammasome activation aids tumorigenesis [85]. Because of the inflammasome complexes' overproduction of IL-1 β and IL-18, a chronic inflammatory state is established, which aids in cancer [86]. IL-1 β and IL-18 are of particular interest in lung cancer because they promote the initiation, progression, and metastasis of the disease. In the lung adenocarcinoma cell line A549, the NLRP3 inflammasome could activate the secretion of IL-18 and IL-1β, the main components of the inflammatory response [87]. 1L-1β and IL-18 are of particular interest in lung cancer because they promote the initiation, progression, and metastasis of the disease [88]. Other studies found that activating the NLRP3 inflammasome promoted breast cancer metastasis to liver and lung tissues [89]. NLRP3 is activated by a wide range of stimuli, whether microbial or sterile [90]. When the NLRP3 inflammasome is activated, mature IL-1 β and IL-18 are produced, resulting in tumor cell infiltration, metastasis, and angiogenesis [91]. IL-1 β produced by NLRP3 promotes cancer cell proliferation and migration in NSCLC by repressing miR-101 via the COX2-HIF1 pathway [92]. IL-1β, on the other hand, has been shown to play a role in the development of the premetastatic niche, and targeting the inflammasome-IL-1β pathway has been proposed as a novel approach for cancer treatment [93]. The activation of the NLRP3 inflammasome pathway is impaired in alveolar macrophages of patients with lung cancer erythematosus (LCE), a rare autoimmune disorder that affects the lungs and other organs [84]. LCE patients have anti-SSA/Ro and anti-SSB/La antibodies that can trigger NLRP3 inflammasome activation and IL-1β secretion in vitro [94]. However, in vivo, their alveolar macrophages exhibit diminished NLRP3, caspase-1 and IL-1β expression, as well as defective inflammasome activation upon LPS and ATP stimulation [95]. This implies that NLRP3 inflammasome may have a beneficial role in LCE by attenuating inflammation and tissue damage. Furthermore, NLRP3 inflammasome may also influence the anti-tumor immune response in LCE patients, as IL-1β can enhance the recruitment and activation of natural killer cells and cytotoxic T cells, which can eliminate tumor cells [22]. Conversely, NLRP3 inflammasome activation can also facilitate lung cancer development and progression by inducing chronic inflammation, angiogenesis, invasion and metastasis [96]. NLRP3 inflammasome can be activated by various stimuli in the lung microenvironment, such as asbestos, silica, cigarette smoke, hypoxia and tumor-derived factors [96]. NLRP3 inflammasome activation results in the production of IL-1β and IL-18, which can stimulate the expression of pro-inflammatory cytokines, chemokines and adhesion molecules, as well as the activation of NF-κB and MAPK signaling pathways [97]. These effects can create a favorable niche for tumor growth and survival, as well as increase the migration and invasion of tumor cells through the modulation of epithelial-mesenchymal transition (EMT) and matrix metalloproteinases (MMPs) [98]. Moreover, NLRP3 inflammasome activation can also promote angiogenesis by inducing the expression of vascular endothelial growth factor (VEGF) and other angiogenic factors in tumor cells and endothelial cells [99]. Additionally, NLRP3 inflammasome activation can suppress the anti-tumor immune response by inducing the polarization of macrophages toward an M2 phenotype, which can secrete immunosuppressive cytokines such as IL-10 and TGF-β, and inhibit the function of T cells and natural killer cells [100]. Therefore, NLRP3 inflammasome has a complex and dual role in lung cancer erythematosus, depending on the context and stage of the disease. Targeting NLRP3 inflammasome or its downstream mediators may have therapeutic potential for LCE patients with lung cancer, but it requires careful consideration of the balance between inflammation and immunity.

### Prostate cancer

In the United States, prostate cancer is the most common cancer and the second leading cause of cancer-related deaths among men [101, 102]. The presence of macrophages and immune suppressor cells was found to be positively associated with the progression of prostate cancer; however, the presence of other cell types with prostate tumors remains inconclusive [101]. The role of pro-inflammatory cytokines and chemokines in facilitating tumor microenvironment and contributing to prostate tumor development is equally important [101]. Clinically, IL-1β, IL-18, IL-6, and MIC-1/GDF-15 levels are associated with the risk of carcinoma and the prognosis of established cancer [103]. Various stimuli, such as urine reflux, uric acid crystals, bacteria, or fungi, are known to cause injury or infection within the prostate. As a result, such stimuli may activate inflammasome-mediated pro-inflammatory cytokines in the prostate, resulting in tumor development [104]. A study of 149 patients found that serum IL-18 levels were significantly higher in locally advanced prostate cancer than in healthy controls [103]. Identifying the regulators of inflammation-associated cytokine/chemokines as a molecular target of cancer progression could lead to a better understanding of the role of inflammation in prostate cancer [101]. Because active caspase-1 facilitates the cleavage of mature IL-1β, it is very likely that by targeting the inflammasome complex, the role of inflammation-associated events in the prostate tumor microenvironment can be manipulated [101]. NLRP3 inflammasome is a key regulator of inflammation in prostate cancer. NLRP3 inflammasome activation in prostate cancer cells can enhance tumor cell growth, survival, migration, and invasion by regulating autophagy, mitochondrial metabolism, and epithelial-mesenchymal transition [105]. NLRP3 inflammasome also triggers the production of IL-1β and IL-18, which stimulate angiogenesis, immune evasion, and metastasis [22]. Moreover, NLRP3 inflammasome can increase the expression of programmed death-ligand 1 (PD-L1) on prostate cancer cells, which inhibits the antitumor immune response mediated by CD8 + T cells [106]. In addition, NLRP3 inflammasome can modulate the recruitment and polarization of tumor-associated macrophages (TAMs) and myeloid-derived suppressor cells (MDSCs), which secrete proinflammatory cytokines and chemokines that support tumor growth and progression [54]. Therefore, targeting NLRP3 inflammasome or its downstream effectors may represent a promising therapeutic approach to inhibit inflammation and improve immunotherapy in prostate cancer.

### Colorectal cancer

**Fig. 3:**
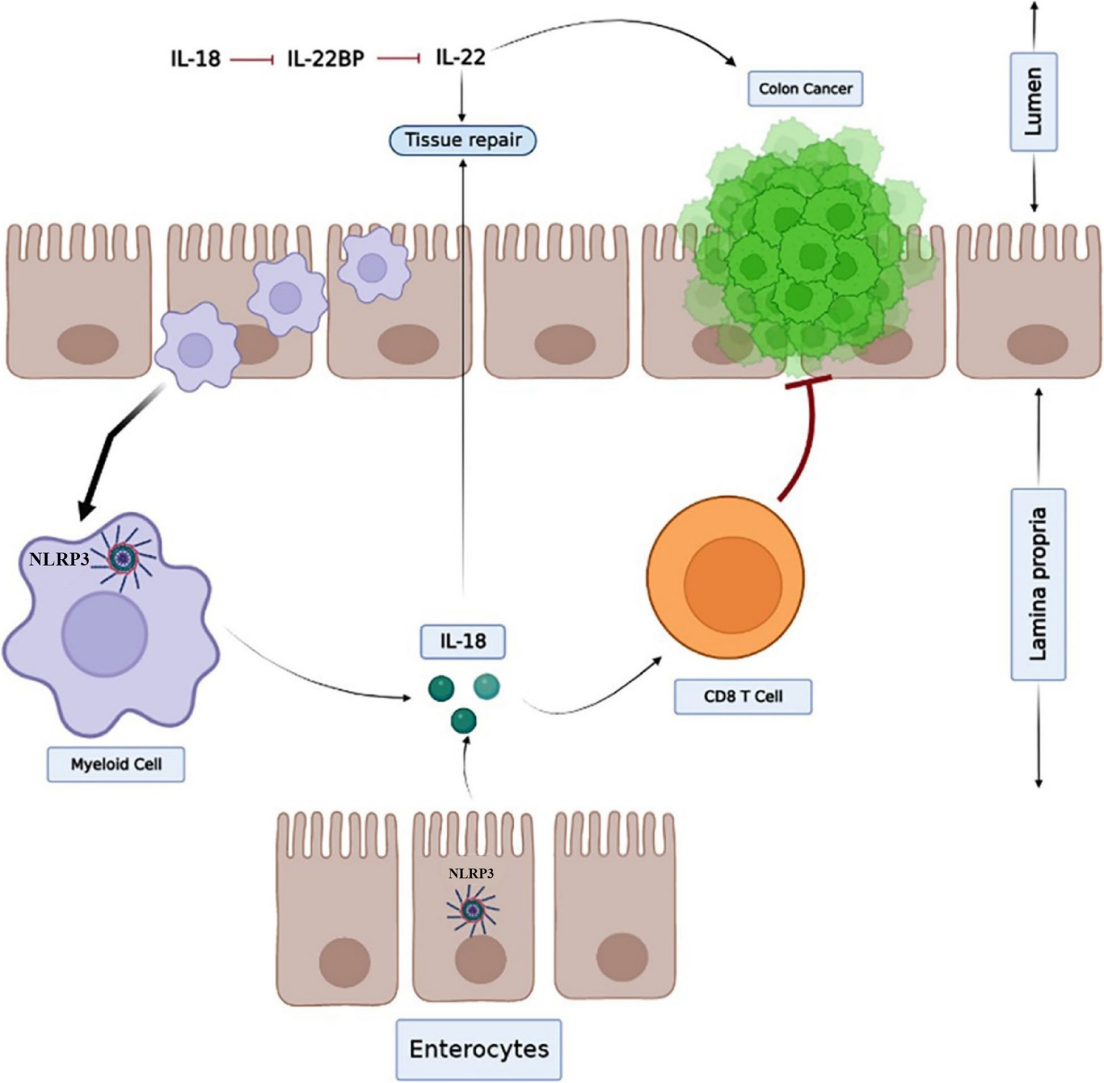
Inflammasomes in (CRC). NLRP3 inflammasome is involved in the release of IL-18 from Kupffer cells, which increases FasL expression on NK cells and promotes their ability to kill tumours. Additionally, IL-18 has been observed to protect against CRC by increasing the epithelial cell barrier and regenerating them. Moreover, IL-22 is modulated by IL-18, with dual effects on CRC

Colorectal cancer (CRC) is one of the most common types of cancer, and it begins as a tissue overgrowth in the rectum or colon [107, 108]. Because of its broad activity in shaping immune response, in vivo and in vitro studies revealed that the NLRP3 inflammasome plays a role in CRC development [107]. The data indicated that NLRP3 was closely related with CRC and may have the capacity to increase CRC invasion and migration, particularly in the advanced stage [109] (Fig. 3).

In colorectal cancer, NLRP3-positive patients had a poor prognosis, and its expression level is closely related to prognosis [110].

It has been reported that inhibiting the activation of NLRP3 inflammasomes can suppress cancer cell inflammation and tumorigenesis [111]. The NLRP3 inflammasome is a critical mediator of inflammation as well as a major regulator of intestinal homeostasis [112].

One proposed mechanism of how CRC progression is influenced by NLRP3 inflammasome activation is that it triggers the secretion of pro-inflammatory cytokines, such as interleukin-1β (IL-1β) and interleukin-18 (IL-18). These cytokines can enhance tumor cell growth, survival, invasion, angiogenesis and immune escape [113, 114]. Pyroptosis, a type of inflammatory cell death, is another mechanism that is activated by NLRP3 inflammasome. This can result in the release of damage-associated molecular patterns (DAMPs) that can amplify the inflammatory response [115]. A third mechanism is that the gut microbiota composition and function, which can influence the intestinal barrier integrity and immune homeostasis, is another factor that is influenced by NLRP3 inflammasome. Furthermore, NLRP3 inflammasome may interact with other signaling pathways, like nuclear factor-κB (NF-κB), hypoxia-inducible factor-1α (HIF-1α) and signal transducer and activator of transcription 3 (STAT3), to modulate CRC development [88, 116]. Therefore, targeting NLRP3 inflammasome may represent a promising strategy for CRC prevention and treatment.

### Bladder cancer

Bladder cancer (BC) is one of the most prevalent urinary system tumors, ranking fourth in terms of male urinary system tumor incidence [117]. Over-activation of nuclear factor-kappa B and STAT signaling, these inflammatory pathways, leading to an aberrant rise in inflammatory cytokines and immune cell over-response, encouraging cancer [118]. According to research, NLRP3 inflammasome pathway dysfunction is linked to a variety of inflammation-induced diseases, and genetic variation in the NLRP3 inflammasome pathway gene is linked to the development of malignant tumors [119]. In one study, they discovered that NLRP3 A/G polymorphisms (rs10754558 and rs35829419) were linked to an increased risk of BC [118]. And They discovered that NLRP3 (rs10754558 and rs35829419) A/G polymorphisms were linked to an increased risk of BC in a study, and their analysis revealed that these relationships were only significant among smokers and drinkers [118]. High expression of the NLRP3 inflammasome is also detected in bladder cancer, making it a possible biomarker for its identification [21]. Furthermore, the activation of NLRP3 inflammasome contributes to the proliferation and dissemination of bladder cancer cells by triggering pyroptosis and releasing IL-1β and IL-18, which enhance angiogenesis, invasion and immune escape [120]. Moreover, NLRP3 inflammasome activation in bladder cancer cells is augmented by hypoxia-inducible factor-1α (HIF-1α), a crucial transcription factor that modulates cellular adaptation to hypoxia, by upregulating the expression of NLRP3 and pro-IL-1β [121]. Therefore, targeting HIF-1α or NLRP3 inflammasome components may represent a novel therapeutic strategy for bladder cancer treatment.

### Melanoma

Melanoma is widely regarded as an immunogenic cancer due to the clearly higher variability in immunogenicity during tumor growth [122]. NLRP3 is required for melanoma growth, progression, and immune response [122]. Reduced inflammasome and IL-1β expression prevented cancer cell development, according to melanoma studies [123]. In a mouse model, thymoquinone treatment reduced metastatic melanoma by downregulating NLRP3 and decreasing IL-1β production [124]. Mice lacking NLRP3, caspase-1, and ASC adaptors are protected from cancer progression [125]. According to one study, NLRP3 expression may influence the composition ratio of B, T cells, and macrophages in the immunological microenvironment of SKCM tumor tissue, therefore indirectly modulating immune monitoring and influencing tumor progression [125]. Additionally, the activation of NLRP3 inflammasome in melanoma cells can promote tumor cell growth and resistance to death by regulating autophagy and mitochondrial energy production [22]. NLRP3 inflammasome also helps tumor cells escape immune recognition by increasing the expression of programmed death-ligand 1 (PD-L1) on melanoma cells, which suppresses the activation and function of CD8 + T cells [106]. Furthermore, NLRP3 inflammasome can influence the attraction and differentiation of tumor-associated macrophages (TAMs), which produce proinflammatory cytokines and chemokines that facilitate tumor growth and blood vessel formation [126]. Therefore, targeting tumor-derived NLRP3 inflammasome may represent a novel therapeutic strategy to enhance antitumor immunity and improve the efficacy of immunotherapy in melanoma.

## Remedial aspects of targeting NLRP3 inflammasome in cancer

However, due to inconsistent results found utilizing tumor cells, the efficiency of inflammasome targeted treatment in malignancies remains unknown [127]. The clinical importance of the NLRP3 inflammasome in several cancers underscores its therapeutic potential as a molecular target [70]. Treatment with a caspase-1 inhibitor and NLRP3 gene silencing inhibited leptin-induced proliferation of breast cancer cells by promoting cell cycle progression and suppressing cell death [128]. According to these findings, using NLRP3 and caspase-1 (MCC950 or Ac-YVAD-cmk) inhibitors to treat breast cancer cells can slow their proliferation [129]. Furthermore, their findings imply that NLRP3 and TLR4 could be novel targets in combination therapy to expand and improve treatment options for BC patients [62]. Although inflammasomes are an important component of the innate immune response, their dysregulation leads to the start, development, and metastasis of lung cancer [21]. An important component of inflammation in our bodies is the inflammasome complex, which converts pro versions of cytokines like IL-1β and IL-18, which aids in cancer progression; thus, investigating various inflammasome inhibitors such as MCC950, CY-09, and others is critical [130]. In recent years, the importance of nucleotide-binding domain and leucine-rich repeat containing receptor (NLR) in carcinogenesis has been discussed, which may lead to novel techniques for CRC treatment. NLRP3 stimulates the tumor-related MAPK signaling pathway during tumor formation and growth, promoting tumor proliferation and migration [131]. These NLRP3 data may show a link between intestinal inflammation and CRC, and may pave the way for a new therapy strategy that targets the NLRP3-MAPK-mTOR-S6K1 axis to improve the prognosis of CRC patients [110]. NLRP3 activates caspase-1, which then cleaves immunological and metabolic substrates, including the pro-inflammatory cytokine interleukin-1b (IL-1β), which causes inflammation and promotes tumor growth. Thus, developing safe and effective NLRP3 inhibitors may benefit in the treatment of CAC (colitis-associated cancer [132]. By targeting NLRP3, miR-22 inhibits cell proliferation, migration, and invasion in colorectal cancer [133]. Several research have revealed The anti-tumor effect of many drugs targeting NLRP3 inflammasomes, including Nigericin and VX-765, was investigated [127]. Nigericin displayed an anti-tumor impact in tumor cell lines with modest NLRP3 stimulation and IL-1β and IL-18 production. In contrast, although Nigericin causes initial tumor cell death in tumor cell lines with strong NLRP3 activation and IL-1β and IL-18 production, cells recover and tumors remain active [134]. Targeting NLRP3 or other downstream signaling molecules, such as caspase-1, IL-1β, or IL-18, offers the potential for significant therapeutic effect [22]. NLRP3 limits antitumor T-cell immunity after dendritic cell vaccination, implying that novel ways to improving responsiveness to anticancer vaccines by restricting NLRP3 signaling are required [135]. The fine-tuning of the NLRP3 inflammasome in cancer cells using a variety of drugs such as inhibitors, antagonists, and monoclonal antibodies has been proposed as a potential cancer therapeutic method [70]. The NLRP3 inflammasome's role in various inflammation-related disorders, including cancer, made it an appealing prospective target for developing novel medications for treatment [21]. Finally, changes in NLRP3 inflammasome activation influence malignant transformation, tumor growth, and therapeutic response through influencing a complicated network of cancer cell activities [70] (Table 1).

**Table 1:** A summary of NLRP3 inflammasome inhibitors effects as potential target in cancer therapy

Type of inhibitor	Target cell/molecule	Interfering molecule	Effect	Result	References
caspase-1 inhibitor	leptin-induced proliferation	Ac-YVAD-cmk	Negative	promoting cell cycle progression and suppressing cell death	[93]
NLRP3 gene silencing	leptin-induced proliferation	MCC950	Negative	promoting cell cycle progression and suppressing cell death	[93]
inflammasome inhibitor	IL-1β and IL-18	CY-09, MCC950	Negative	aids in cancer progression	[94]
NLRP3 inhibitor	NLRP3	miR-22	Positive	inhibits cell proliferation, migration, and invasion	[97]
NLRP3 inhibitor	NLRP3 inflammasomes	VX-765	Positive	anti-tumor effect by modest NLRP3 activation and IL-1β and IL-18 production	[95]

## Protective roles of inflammasomes in cancer

NLRP3-dependent IL-18 production inhibits neoplastic events, potentially by inducing IFN-γ production and STAT1 signaling [136]. NLRP3 is also involved in cancer prevention in the liver [77]. Furthermore, in human liver cancer tissues, the expression of NLRP3 inflammasome components is drastically downregulated or entirely eliminated [137]. The overexpression of NLRP3 by 17β-estradiol (E2) inhibits the development of hepatocellular carcinoma cells, indicating that NLRP3 may play a protective function in hepatic malignancy [138]. The data imply that NLRP3 plays a tumor suppressor role in colorectal cancer [22]. The NLRP3 inflammasome appears to be a negative tumorigenesis modulator in colitis-associated cancer [23]. Mice lacking IL-1R and caspase-1 showed partial protection against skin cancer development in epithelial skin carcinoma [21]. Dendritic cell-mediated priming of IFN-γ-producing T lymphocytes against tumor cells requires the NLRP3 inflammasome [125]. Ultimately The role of the NLRP3 inflammasome in human malignancies remains a contentious issue [21].

## Predisposing roles of NLRP3 in cancer

In humans, NLRP3 inflammasome signaling is influenced by a range of factors, including genetic polymorphisms and mutations that change gene expression and, eventually, contribute to its activation. These effects were observed in people suffering from inflammatory disorders [21]. Similarly, NLRP3 inflammasome genetic variants have been associated to cancer [21]. Changes in the NLRP3 gene's sequence could affect its activity. The NLRP3 gene has approximately 60 single nucleotide polymorphisms (SNPs). rs4612666 and rs10754558 and Q705K (rs35829419), which are situated in the 3'-UTR of the NLRP3 gene and may impact the stability and expression of NLRP3 mRNA, have been widely researched [21, 139]. These two SNPs have been linked to a variety of multifactorial diseases, including coronary artery disease and cancers such as Philadelphia chromosome-negative myeloproliferative neoplasms and lung cancer, but their significance in the development of Bladder Cancer remains unknown [140]. rs35829419 was correlated with poorer survival in patients with invasive colorectal cancer, postulated as a risk allele for sporadic metastatic melanoma in Swedish males, and also occurs at high frequency in pancreatic cancer patients [141]. NLRP3 inflammasome pathway failure has been linked to a variety of inflammation-induced disorders, and genetic variation in the NLRP3 inflammasome pathway gene has been linked to the development of malignant tumors such as chronic myeloid leukemia and melanoma [119]. Only IL-1 and IL-18 polymorphisms in hematological malignancies were linked to clinical and pathophysiological traits in acute myeloid leukemia (AML) and chronic myeloid leukemia (CML). Additionally, research using gene expression profiling has linked the activation of the NLRP3 inflammasome to a number of malignancies. In Head and neck squamous cell carcinoma (HNSCC) Laryngeal squamous cell carcinoma ( LSCC), and squamous cell carcinoma tissues, for instance, NLRP3 is overexpressed in comparison to normal tissues; this overexpression is frequently associated with a poor prognosis and worse pathology [142]. NLRP3 may also influence tumor responses by interfering with the efficacy of immunotherapy [143]. Furthermore, NLRP3 inflammasome dysfunction appears to increase tumor burden in colorectal cancer [144]. NLRP3 is essential for caspase-1 activation and the release of the pro-inflammatory cytokines IL-1β and IL-18 [145]. More research is needed to understand the relationship between genetic variants or variable expression of the NLRP3 inflammasome and cancer clinical characteristics.

## Conclusion

NLRP3 plays double-edged sword in carcinogenesis, exhibiting both negative and positive effects. Only in the context of CAC and some kinds of liver cancer43 has the positive effect been clearly documented. However, negative effects have been reported in various malignancies and metabolic illnesses such as diabetes, obesity, and atherosclerosis. Inflammasome effector cytokines, IL-1β and IL-18, are important effector molecules that aggravate these disorders when NLRP3 is activated. The function of NLRP3 in specific tumors may also be affected by the effects of other mutations on its expression, tumor type, tumor stage, and effector molecules downstream of NLRP3. As a result, NLRP3 signaling has complicated consequences on tumor start and development through modulating antitumor immunity, cell death, proliferation, angiogenesis, and metastasis. Given that the effects of inflammasome signaling differ between tumor types, understanding how to regulate this variability will be a key topic of future research. As a result, by examining the functional pathways and how NLRP3 works in different types of cancer, with more investigations and more detailed studies, this mechanism can be used in the treatment of different types of cancer, although according to the data obtained, this use is not the same in the treatment of different types of cancer.

### Supplementary Information

**Additional file 1.**

## Data Availability

Not applicable.
