# Supplementary material for: Divergent functions of NLRP3 inflammasomes in cancer: a review
Source: Cell Commun Signal. 2023 Sep 15;21:232. doi: 10.1186/s12964-023-01235-9 (PMC10503066; doi:10.1186/s12964-023-01235-9)
Supplement: Supplementary file 2 — Additional file 1. [file 12964_2023_1235_MOESM1_ESM.pdf]

SPRINGER NATURE

Change of authorship request form - Journals (pre-acceptance)

Important information. Please read.

- This form should be used by authors to request any change in authorship (adding/deleting authors) including changes in corresponding authors. This form should not be used for name changes. Please fully complete all sections. Use black ink and block capitals and provide each author's full name with the given name first followed by the family name.

- By signing this declaration, all authors guarantee that the order of the authors are in accordance with their scientific contribution, if applicable as different conventions apply per discipline, and that only authors have been added who made a meaningful contribution to the work.

- Please note, in author collaborations where there is formal agreement for representing the collaboration, it is sufficient for the representative or legal guarantor (usually the corresponding author) to complete and sign the Authorship Change Form on behalf of all authors, **next to the added/removed author(s)**. **(Complete Section 3, followed by Section 6.)**
  In author collaborations where there is no formal agreement for representing the collaboration and **there are more than 10 authors**, one may sign for all, provided the signer appends correspondence that attests that each of the authors have agreed to the change **and the added/removed authors sign the form. (Complete Section 3, followed by Section 6.)**

- Please note, we cannot investigate or mediate any authorship disputes. If you are unable to obtain agreement from all authors (including those who you wish to be removed) you must refer the matter to your institution(s) for investigation. Please inform us if you need to do this.

- If you are not able to return a fully completed form within **30 days** of the date that it was sent to the author requesting the change, we may have to withdraw your manuscript. We cannot publish manuscripts where authorship has not been agreed by all authors (including those who have been removed).

- Incomplete forms will be rejected.

- Please return/upload this form, fully completed, to the Journals Editorial Office. The Journal and/or Publisher will consider the information you have provided to decide whether to approve the proposed change in authorship. We may decide to contact your institution for more information or undertake a further investigation, if appropriate, before making a final decision.

Scanned with CamScanner

SPRINGER NATURE

Change of authorship request form - Journals (pre-acceptance)

Section 1: Please provide the current title of manuscript

Manuscript ID no.:

Title: Divergent functions of NLRP3 inflammasomes in cancer: A review

Section 2: Please provide the previous authorship, in the order shown on the manuscript before the changes were introduced. Please indicate the corresponding author by adding (CA) behind the name.

	First name(s)	Family name	ORCID or SCOPUS id, if available
1st author	ALIREZA	SHADAB	0000-0003-2404-9872
2nd author	MOHAMMAD*	MAHJOOR*	0000-0001-6857-0323
3rd author	MOHAMMAD	ABBASI-KOLLI	0000-0003-2661-9613
4th author	HAMED	AFKHAMI	0000-0002-1110-6447
5th author	AMIR-REZA (CA)	SAFDARIAN (CA)	0009-0005-3477-9088
6th author			
7th author			
8th author			
9th author			
10th author			

Please use an additional sheet if there are more than 10 authors.

*Co-First Author (ALIREZA SHADAB - MOHAMAD MAHJOOR)

SPRINGER NATURE Springer Nature is one of the world's leading global research, educational and professional publishers, created in May 2015 through the combination of Nature Publishing Group, Palgrave Macmillan, Macmillan Education and Springer Science+Business Media.

Scanned with CamScanner

SPRINGER NATURE

Change of authorship request form - Journals (pre-acceptance)

Section 3: Please provide a justification for change. Please use this section to explain your reasons for changing the authorship of your manuscript, e.g. what necessitated the change in authorship? Please refer to the (journal) policy pages for more information about authorship. Please explain why omitted authors were not originally included and/or why authors were removed on the submitted manuscript.

After reviewing the article and submitting the reviewers comments, we added one author (parisa Moeinian) to the last place of our article to help us with the review

Section 4: Proposed new authorship. Please provide your new authorship list in the order you would like it to appear on the manuscript. Please indicate the corresponding author by adding (CA) behind the name. <u>If the Corresponding Author has changed, please indicate the reason under section 3</u>.

	First name(s)	Family name (this name will appear in full on the final publication and will be searchable in various abstract and indexing databases)	Affiliated institute	E-mail address
1st author	ALIREZA	SHADAB	Semnan University of Medical Sciences	shadab.alireza@gmail.com
2nd author	MOHAMAD	MAHJOOR	Qom University of Medical Sciences	m.mahjoor2118@gmail.com
3rd author	MOHAMMAD	ABBASI-KOLLI	Tarbiat Modares University	m.abbasi.gene@gmail.com
4th author	HAMED	AFKHAMI	Semnan University of medical sciences	hamedafkhami70@gmail.com
5th author	PARISA	MOEINIAN	Iran University of Medical sciences	moeini92.pm@gmail.com
6th author	AMIR-REZA	SAFDARIAN (CA)	USERN Chicago	Safdarian.amir.r@ut.ac.ir
7th author				
8th author				
9th author				
10th author				

Please use an additional sheet if there are more than 10 authors.

SPRINGER NATURE Springer Nature is one of the world's leading global research, educational and professional publishers, created in May 2015 through the combination of Nature Publishing Group, Palgrave Macmillan, Macmillan Education and Springer Science+Business Media.

Scanned with CamScanner

Change of authorship request form - Journals (pre-acceptance)

Section 5: Author contribution, Acknowledgement and Disclosures. Please use this section to provide a new disclosure statement and, if appropriate, acknowledge any contributors who have been removed as authors and ensure you state what contribution any new authors made (if applicable per the journal or book (series) policy). Please ensure these are updated in your manuscript - after approval of the change(s) - as our production department will not transfer the information in this form to your manuscript.

New acknowledgements:

Not applicable

New Disclosures (financial and non-financial interests, funding):

NO Funding

New Author Contributions statement (if applicable per the journal policy):

PARISA MOEINIAN (PM): edited manuscript
(Of course, in the revised version of the article that we have submitted to your prestigious journal, we have added her name to contributions statement section)

State 'Not applicable' if there are no new authors.

Scanned with CamScanner

SPRINGER NATURE

Change of authorship request form - Journals (pre-acceptance)

Section 6: Declaration of agreement. ***All*** **authors, unchanged, new and removed** ***must*** **sign this declaration.**
(NB: Please print the form, (docu)-sign and return/upload a scanned copy. Please note that signatures that have been inserted as an image file are acceptable as long as it is handwritten. Typed names in the signature box are unacceptable.) *** Please delete as appropriate. Delete all of the bold if you were on the original authorship list and are remaining as an author.**

	First name	Family name		Signature	Date
1st author	ALIREZA	SHAPAB	I agree to the proposed new authorship shown in section 4 ~~**/and the addition/removal*of my name to the authorship list**~~ /and the proposed change in corresponding author		2023/7/6
2nd author	MOHAMAD	MAHJOOR*	I agree to the proposed new authorship shown in section 4 ~~**/and the addition/removal*of my name to the authorship list**~~ /and the proposed change in corresponding author		2023/7/6
3rd author	MOHAMMAD	ABBASI-KOLLI	I agree to the proposed new authorship shown in section 4 ~~**/and the addition/removal*of my name to the authorship list**~~ /and the proposed change in corresponding author		2023/7/6
4th authors	HAMED	AFKHAMI	I agree to the proposed new authorship shown in section 4 ~~**/and the addition/removal*of my name to the authorship list**~~ /and the proposed change in corresponding author		2023/7/6
5th author	PARISA	MOEINIAN	I agree to the proposed new authorship shown in section 4 **/and the addition/~~removal*~~ of my name to the authorship list** /and the proposed change in corresponding author	PARISA	2023/7/6
6th author	AMIR-REZA	SAFDARIAN	I agree to the proposed new authorship shown in section 4 ~~**/and the addition/removal*of my name to the authorship list**~~ /and the proposed change in corresponding author		2023/7/6
7th author			I agree to the proposed new authorship shown in section 4 **/and the addition/removal*of my name to the authorship list** /and the proposed change in corresponding author		

* Co-First Author (ALIREZA SHAPAB – MOHAMAD MAHJOOR)

SPRINGER NATURE Springer Nature is one of the world's leading global research, educational and professional publishers, created in May 2015 through the combination of Nature Publishing Group, Palgrave Macmillan, Macmillan Education and Springer Science+Business Media.

Scanned with CamScanner

Change of authorship request form - Journals (pre-acceptance)

	First name	Family name		Signature	Date
8th author			I agree to the proposed new authorship shown in section 4 **/and the addition/removal*of my name to the authorship list** /and the proposed change in corresponding author		
9th author			I agree to the proposed new authorship shown in section 4 **/and the addition/removal*of my name to the authorship list** /and the proposed change in corresponding author		
10th author			I agree to the proposed new authorship shown in section 4 **/and the addition/removal*of my name to the authorship list** /and the proposed change in corresponding author		

Please use an additional sheet if there are more than 10 authors.

In case of author collaborations with formal agreement:

	Name of consortium/consortia	First name	Family name		Signature	Date
Representative/legal guarantor		AMIR-REZA	SAFDARIAN	I agree to the proposed new authorship shown in section 4 **/and the addition/removal*of my name to the authorship list** /and the proposed change in corresponding author	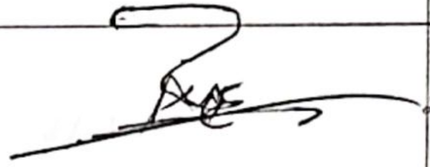	2023,7,6

Both added/removed authors should complete the information in the first table under Section 6.

---- End of form ----

SPRINGER NATURE Springer Nature is one of the world's leading global research, educational and professional publishers, created in May 2015 through the combination of Nature Publishing Group, Palgrave Macmillan, Macmillan Education and Springer Science+Business Media.

Scanned with CamScanner
